# Optimizing board structure for ESG integrity: Nonlinear size effects and diversity moderation on greenwashing

**DOI:** 10.1371/journal.pone.0335803

**Published:** 2026-01-23

**Authors:** Jingzhuo Yu, Yong-Sik Hwang

**Affiliations:** 1 School of Civil Engineering and Architecture, Jiaxing Nanhu University, Jiaxing, Zhejiang, China; 2 School of Business and Economics, Sejong University, Seoul, Republic of Korea; University of Almeria: Universidad de Almeria, SPAIN

## Abstract

This study examines the nonlinear relationship between board size and Environmental, Social, and Governance (ESG) greenwashing and explores how board diversity moderates this association. Using panel data from Chinese A-share listed firms between 2009 and 2023, we employ quadratic fixed-effects regression models to test for an inverted U-shaped relationship. The results indicate that medium-sized boards (10–13 directors) exhibit the highest propensity for greenwashing. Further analyses reveal heterogeneous moderating effects across four dimensions of board diversity—gender, functional background, nationality, and age. Specifically, reaching a critical mass of at least two female directors or increasing functional diversity strengthens the inverted U-shaped relationship, whereas greater age or nationality diversity attenuates it. Drawing on fraud triangle theory, this study uncovers a previously overlooked nonlinear mechanism underlying board size and ESG greenwashing. Moreover, it identifies two distinct diversity-driven pathways: resource-based mechanisms (gender and functional diversity) and supervision-based mechanisms (nationality diversity). These findings extend existing literature on board governance and greenwashing and provide practical insights, suggesting that firms should avoid the “danger zone” associated with medium-sized boards and adopt targeted diversity strategies to mitigate ESG greenwashing risks.

## 1. Introduction

Corporate sustainability reporting has surged worldwide, yet concerns about “greenwashing” —the gap between firms’ environmental rhetoric and reality—are at an all-time high. Fully 85% of institutional investors now view greenwashing as a worsening problem [1]. This paradox is especially evident in China, where regulators have aggressively promoted Environmental, Social and Governance (ESG) disclosure through mandatory reporting requirements for companies in major indices and those with international listings. In April 2024, China’s three major stock exchanges—Shanghai, Shenzhen, and Beijing—jointly implemented ESG Reporting Guidelines, establishing mandatory disclosure requirements for companies listed in prominent indices (SSE 180, STAR 50, SZSE 100, and ChiNext), as well as firms maintaining both domestic and international listings. More than 2,400 Chinese A-share companies released ESG reports in 2024, accounting for approximately 46.09% of all A-share listed firms [2], but officials felt compelled to warn that “ESG reports are not advertisements”—a telling sign that many firms’ sustainability claims may be more cosmetic than substantive [3,4]. Such misrepresentations not only erode stakeholder trust and misallocate capital, but also undermine genuine environmental progress and corporate legitimacy in the long run [5–7].

The urgency of this issue raises the question of how corporate governance can curb ESG greenwashing in practice and in theory. Boards of directors serve as a frontline of oversight, responsible for ensuring truthful disclosure and ethical behavior [8]. Prior research suggests that certain board attributes can restrain greenwashing—for example, more female directors or independent directors tend to engage in less greenwashing [9,10]. However, the influence of board size remains contested and underexplored. Some scholars, drawing on resource dependence theory [11], argue that larger boards possess broader expertise and networks, thereby enhancing oversight and transparency [12]. In contrast, others, based on agency theory [13], emphasize the coordination inefficiencies associated with oversized boards [14]. Empirical findings to date are mixed: one recent study found no clear link between board size and greenwashing [15], while another found a positive relationship between these variables [16], reflecting a broader gap in understanding which board structures truly deter ESG misrepresentation. Notably, prior studies have treated board size effects as linear and direct; few have considered the possibility of a non-linear relationship [17,18]. Furthermore, most existing studies examining the relationship between board characteristics and greenwashing have predominantly focused on gender diversity and board independence, often through the lens of agency theory [19,20]. These studies may emphasize the role of such characteristics in enhancing oversight to curb greenwashing but tend to overlook an equally important function—resource provision [21,22]. Three critical questions therefore remain unanswered:

**RQ1**: Does board size exhibit a non-linear relationship with ESG greenwashing?

**RQ2**: Can the dual mechanisms of oversight and resource provision jointly explain this relationship?

**RQ3**: How do multiple dimensions of board diversity—beyond gender alone—moderate the board size–greenwashing relationship?

To address these questions, the present study integrates fraud triangle theory (FTT) with both agency and resource dependence perspectives, examining how board size relates to greenwashing through opportunity and pressure mechanisms, and how four diversity dimensions (gender, functional background, age, and nationality) condition this relationship.

To achieve the above research objectives, this study analyzes panel data from Chinese listed companies (2009–2023). China is chosen as the research context due to its unique institutional environment—characterized by underdeveloped ESG disclosure standards, a lack of anti-greenwashing regulations [23–25], a capital market dominated by retail investors with significant information disadvantages [26], and the dual effects of institutional investors on corporate social responsibility (CSR) reports [27]. These factors not only create greater opportunities for greenwashing but also provide a rich empirical setting for examining the complex relationship between board characteristics and ESG greenwashing behavior, thereby revealing governance mechanisms that may be generalizable to other developing economies.

This study contributes to the literature on board governance and greenwashing in at least three ways. First, we advance greenwashing research by moving beyond the prevailing linear, performance-focused perspective to theorize and test a nonlinear relationship between board size and greenwashing. Prior work grounded in agency and resource dependence theories has reported mixed linear associations between board size and ESG/CSR performance—including negative, positive, inverted U-shaped, and insignificant effects—while only a few studies explicitly examine greenwashing [28–30]. Focusing on ESG greenwashing and drawing on FTT, we develop an opportunity–pressure mechanism that predicts, and our evidence confirms, an inverted U-shaped relationship between board size and greenwashing. This finding helps reconcile prior inconsistencies and demonstrates that board size is neither uniformly beneficial nor uniformly harmful, but shapes ESG-related misconduct in a nonlinear way.

Second, we refine and extend research on the nonlinear effects of board size by challenging the notion of a single “optimal” board size. Recent studies, such as Papadopoulou et al. (2025), identify an inverted U-shaped relationship between board size and CSR or sustainability performance and argue that there exists a performance-maximizing size [31]. In contrast, our study reveals an opposite pattern: when ESG greenwashing is the focal outcome, we find an inverted U-shaped relationship, where both relatively small and relatively large boards are associated with lower levels of greenwashing, whereas medium-sized boards exhibit the highest propensity for symbolic ESG disclosure. Thus, instead of a universal optimal size, our results suggest multiple effective board size configurations depending on governance objectives and the trade-off between symbolic and substantive ESG engagement.

Third, this study enriches the board diversity literature from a fine-grained, mechanism-based perspective. Prior research, including recent work by Papadopoulou et al. (2025), typically examines only gender diversity as a moderator of board size effects, leaving other diversity dimensions underexplored [31]. Moreover, most studies focus on the linear main effects of diversity on ESG greenwashing or CSR decoupling, rather than examining how diversity conditions nonlinear governance relationships [32]. In contrast, we decompose board diversity into four dimensions—gender, functional background, age, and nationality—and embed them in an FTT-inspired opportunity–pressure framework. This design allows us to distinguish supervision-based from resource-based mechanisms and to show how each diversity dimension differentially reshapes the board size–greenwashing relationship.

The remainder of this paper is organized as follows: Section 2 reviews the relevant literature and discusses the theoretical foundation of this study. Section 3 presents the research hypotheses. Section 4 introduces the methodological framework. Section 5 presents the empirical results, including the baseline regression analysis, robustness tests, and moderation effect analysis. Finally, Section 6 discusses and analyzes the main conclusions, highlights their theoretical and practical implications, and points out the limitations of the study.

## 2 Literature review

### 2.1 Greenwashing

The concept of greenwashing has evolved from product-level deceptive environmental claims [33], to CSR decoupling [34], and more recently to applications within the ESG framework [35]. At the firm level, ESG greenwashing is typically understood as a systematic discrepancy between symbolic disclosure and substantive performance [36]. Companies engaging in greenwashing strategically manipulate ESG information to project an inflated image of environmental and social responsibility while obscuring weak underlying performance [37–39]. This “words-versus-actions” gap represents a form of strategic decoupling, whereby firms invest disproportionately in ESG communication and disclosure activities relative to their actual ESG implementation efforts [10]. Consistent with this view, ESG greenwashing is usually measured as the gap between an ESG disclosure score and an ESG performance rating [24]. Disclosure scores, such as Bloomberg’s ESG disclosure score, primarily capture the extent and intensity of publicly reported ESG information, whereas performance ratings, such as Huazheng’s ESG performance rating, reflect underlying ESG outcomes and practices. A larger positive gap therefore indicates greater reliance on symbolic ESG disclosure relative to substantive performance—i.e., more severe greenwashing—whereas negative values may signal “greenhushing,” where firms disclose conservatively despite relatively strong ESG performance [40].

### 2.2 Fraud triangle theory

Greenwashing can be conceptualized as a form of fraudulent behavior in corporate information disclosure, due to its structural similarities with fraud [41]. Consequently, FTT, which identifies three core motivations behind fraud—pressure, opportunity, and rationalization—offers a valuable analytical framework for exploring ESG greenwashing [42]. Pressure in this context typically manifests as legitimacy pressure, driven by the need to fulfill stakeholder expectations or regulatory demands [43]. Firms with poor ESG performance face heightened legitimacy risks, which motivate them to alleviate stakeholder concerns by exaggerating or fabricating ESG achievements, thus transforming disclosure from a legitimizing tool into a greenwashing tactic [44,45]. Opportunity relates to the likelihood of engaging in greenwashing without detection, facilitated by information asymmetry and weak supervision [46]. One of the most classic examples of creating opportunity through information asymmetry is by reducing the readability of ESG reports, to obscure negative details and diminish stakeholder responsiveness [47]. Weak oversight from internal governance structures or external monitors such as institutional investors, analysts, auditors, and media further creates greenwashing opportunities [48]. Finally, rationalization involves internally justifying unethical actions by framing them as legitimate strategies [49]. For example, the greenwashing “herding effect” triggered by regional isomorphism [50] may be rationalized by firms as a “common survival strategy within the industry.” This cognitive justification reduces ethical discomfort and fosters an environment where misleading ESG disclosures become psychologically acceptable. Recent studies successfully apply the FTT to greenwashing research [51,52].

### 2.3 Board size

Board size has a significant impact on governance effectiveness [53], yet existing research offers two conflicting perspectives. From an agency theory perspective, smaller boards enhance governance efficiency and oversight because they facilitate closer collaboration, clearer accountability, and more effective monitoring of management [13]. By contrast, larger boards are more prone to bureaucracy, coordination difficulties, and reduced cohesion, which can lower efficiency and weaken monitoring [54], thereby creating conditions conducive to greenwashing. Empirical evidence supports these adverse effects, documenting a negative association between board size and ESG performance [30], a negative association with governance performance [29], and a positive association with ESG greenwashing [16].

Resource dependence theory, however, emphasizes the advantages of larger boards, including broader expertise, greater access to critical resources, and richer stakeholder networks [11]. These attributes enable boards to respond more effectively to diverse stakeholder demands, strengthen ethical practices, improve ESG or CSR performance, and reduce legitimacy pressures, thereby lowering incentives to engage in greenwashing. Empirical studies show that firms with larger boards tend to exhibit higher ESG performance [55,56], better CSR performance [28], improved long-term environmental performance [57], fewer ESG controversies [58,59], and stronger ethical business practices [60].

Several nuanced patterns further complicate this debate. Some evidence suggests that the effect of board size is context-dependent: in China, board size is positively associated with CSR performance, whereas in India and South Africa it is negatively associated [61]. Other work documents an inverted U-shaped relationship between board size and ESG/CSR performance [31,62]. Finally, some studies report no significant linear relationship between board size and ESG performance [29] or between board size and greenwashing [15].

Notably, among these studies, only Gidage et al. and Keresztúri et al. explicitly examine ESG greenwashing [15,16], while Agnese et al. and Treepongkaruna et al. focus on ESG controversies [58,59]. The remaining studies investigate overall ESG or CSR performance rather than misconduct or disclosure-performance gaps. Consequently, while prior research emphasizes linear relationships between board size and ESG/CSR outcomes, few studies directly address greenwashing, and virtually none theorize or test non-linear effects. Our study addresses this gap by explicitly focusing on ESG greenwashing and developing a non-linear board-size mechanism grounded in FTT.

### 2.4 Diversity

Diversity can be categorized into relational-oriented dimensions (including “surface-level” differences such as gender, race, and age) and task-oriented dimensions (including “deep-level” or job-related differences such as tenure and expertise) [63]. This study examines four dimensions—gender, functional background, nationality, and age—covering these diversity types. Boards primarily serve supervisory and advisory roles [21]. From a supervisory standpoint, diversity promotes critical thinking, reduces groupthink, enhances independence, and enriches oversight [64]. However, excessive diversity may cause factionalism (“Faultline” effect), reduce cohesion, and increase conflicts, weakening supervision [65,66]. From an advisory perspective, diverse boards offer extensive expertise and external networks, enhancing responsiveness to stakeholders and improving ESG and CSR outcomes [67,68]. Conversely, social identity theory suggests diversity can negatively impact performance due to higher internal conflicts, coordination costs, and reduced consensus [69,70]. In summary, board diversity represents a “double-edged sword” [71], with its effects varying based on supervisory and advisory contexts [72]. Additionally, impacts differ according to environmental conditions, institutional contexts, and diversity definitions, highlighting the need for focused, context-specific research [73,74].

## 3 Hypotheses development

### 3.1 Board size and ESG greenwashing

**Fig 1:**
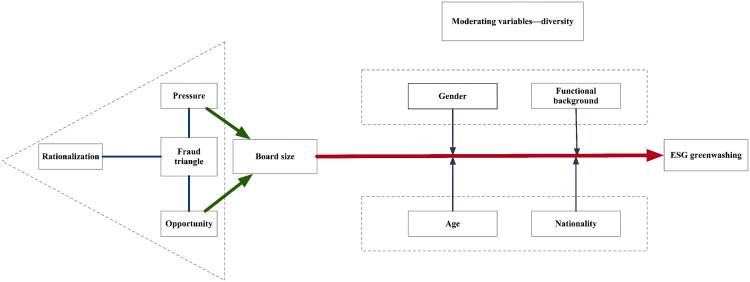
Theoretical model framework.

The theoretical model framework is shown in Fig 1. Building on FTT, ESG greenwashing can be understood as resulting from three primary psychological and structural drivers: opportunity, pressure, and rationalization [75]. While “rationalization” is often treated as relatively stable and implicit [48], “opportunity” and “pressure” can vary substantially across firms depending on factors such as board structure. Drawing upon Haans et al. (2016), an inverted U-shaped relationship may emerge when two underlying linear mechanisms—one positive and one negative—interact multiplicatively [76]. This approach is particularly applicable when an outcome, typically a strategic choice such as greenwashing, is influenced simultaneously by two opposing forces [76]. They illustrate this concept through examples such as Ang (2008), who found that corporate collaboration is most frequent at moderate levels of competitive intensity [77]. Specifically, Ang (2008) identified an inverted U-shaped relationship between competitive intensity and collaboration [77], constructed by multiplying a negative linear function (decreasing opportunity for collaboration with increasing competitive intensity) and a positive linear function (increasing motivation for collaboration with increasing competitive intensity).

Applying this theoretical framework to ESG greenwashing, we propose that board size simultaneously influences the opportunity and pressure dimensions. As board size grows, the potential for greenwashing may increase due to weakened oversight capabilities, heightened information asymmetry, and coordination inefficiencies [53]. On the other hand, larger boards typically offer broader expertise and more extensive external networks, enabling firms to enhance their stakeholder management capabilities and achieve substantive improvements in ESG practices, thus potentially alleviating legitimacy pressures [78].

**Fig 2:**
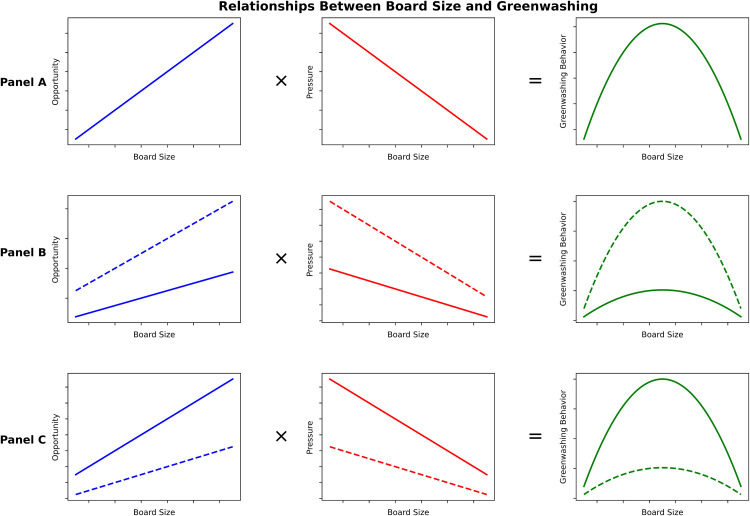
Latent mechanism of the inverted U-shaped relationship. Note: Solid lines indicate the pre-change state; dashed lines indicate the post-change state.

Consequently, corporate greenwashing behaviors can be considered the result of an interplay between opportunity and pressure. Small boards may experience higher legitimacy pressure due to limited resources and weaker genuine ESG capabilities, though their oversight strength reduces management’s greenwashing opportunities [79]. In contrast, larger boards may provide more opportunities for greenwashing due to diluted oversight but have access to resources that can facilitate genuine ESG improvements and thus mitigate legitimacy pressures [20]. Hence, moderately-sized boards experience intermediate levels of both opportunity and pressure. The interplay between these two factors—opportunity × pressure—likely peaks when both elements are moderate, creating conditions for the highest risk of ESG greenwashing. This interaction aligns with the “Type 3” interaction structure outlined by Haans et al. (2016) [76], where positive and negative linear relationships interact to produce an inverted U-shaped effect. This conceptualization is visually depicted in Panel A of Fig 2 and informs the following hypothesis:

H1: There is an inverted U-shaped relationship between board size and ESG greenwashing.

### 3.2 The moderating effect of diversity

We suggest that the inverted U-shaped relationship between board size and ESG greenwashing may arise from the interaction between opportunity and pressure factors. Board diversity may moderate this relationship by affecting how rapidly opportunities increase and pressures decrease as board size expands. We identify four possible moderation mechanisms: (i) both opportunity and pressure changes accelerate; (ii) opportunity increases accelerate while pressure decreases decelerate; (iii) opportunity increases decelerate while pressure decreases accelerate; and (iv) both opportunity and pressure changes decelerate. Mechanism (i) potentially steepens the inverted U-shaped curve (as shown in Panel B of Fig 2), whereas mechanism (iv) flattens it (Panel C of Fig 2). For mechanisms (ii) and (iii), outcomes may depend on the dominant effect between opportunity and pressure. In the following sections, we explore the moderating effects of diversity along four dimensions: gender, functional background, nationality, and age.

#### 3.2.1 Gender diversity.

From the perspective of FTT, gender diversity can mitigate both pressure and opportunity for greenwashing, but through partly distinct mechanisms. Drawing on gender role theory, female directors are often socialized into more communal and prosocial roles than men, emphasizing care, empathy, and relationship building [80,81]. On boards, this tends to heighten sensitivity to the “Social” pillar of ESG (e.g., employee welfare, community relations, stakeholder inclusion) and to fairness, transparency, and procedural justice within the “Governance” pillar [82]. These role-consistent traits enrich the board’s relational and reputational resources—such as trust with employees, communities, and NGOs—and enhance its capacity to anticipate and address social and environmental concerns [83].

From a gender equity perspective, greater female representation also signals a commitment to equality and inclusion that is increasingly expected by regulators, investors, and civil society. Gender-diverse boards are therefore more likely to be perceived as legitimate and socially responsible, which can reduce external legitimacy pressures that might otherwise induce symbolic ESG communication and greenwashing [84]. By aligning board composition with societal and stakeholder expectations, gender diversity can pre-empt reputational criticism related to both diversity and ESG conduct, thereby easing pressure to resort to impression management. In this way, female directors play a resource-provision role by bringing distinct perspectives, stakeholder-oriented values, and legitimacy-enhancing signals that help boards manage ESG-related pressures more effectively.

Gender diversity also affects the opportunity side of greenwashing. Prior research shows that female directors tend to exhibit higher diligence, ethical sensitivity, and risk aversion, and are more likely to challenge questionable managerial behavior [85,86]. Their presence can strengthen monitoring intensity, improve the quality of board deliberations, and limit managers’ ability to exploit information asymmetries to misrepresent ESG performance [87,88]. Thus, gender-diverse boards function as a governance mechanism that constrains opportunities for misleading ESG disclosures and opportunistic greenwashing.

Overall, gender diversity may exert dual effects on the inverted U-shaped relationship between board size and greenwashing. First, female directors expand the board’s resource base—particularly stakeholder insight and reputational capital—thereby steepening the inverted U-shaped curve. Second, by enhancing oversight and ethical scrutiny, gender diversity may dampen greenwashing opportunities in larger boards, flattening the relationship. This represents a type (iii) among the four moderation mechanism combinations. While female directors’ resource-provision function may be salient under strong legitimacy pressures, their monitoring function is equally important. Theory does not clearly predict which mechanism will dominate ex ante. Thus, we propose a non-directional hypothesis:

H2. Gender diversity moderates the inverted U-shaped relationship.

#### 3.2.2 Functional background diversity.

Functional background diversity may also help mitigate the two critical elements of the fraud triangle: pressure and opportunity. In terms of pressure, boards with functional diversity contribute specialized knowledge, skills, and external networks [89], and demonstrate stronger capabilities in identifying and responding to various social and environmental challenges [28]. This enables them to better understand and coordinate the interests of diverse stakeholders [90], thereby enhancing stakeholder management and promoting more proactive ESG actions [91], which effectively alleviates the legitimacy pressures that often drive greenwashing behavior. Regarding opportunity, functional diversity creates a “collective intelligence” effect that improves board oversight and reduces the risk of managerial manipulation through information asymmetry [63,92], thus limiting opportunities for misleading ESG disclosures.

Therefore, we posit that functional diversity may simultaneously exert dual effects: on the one hand, by providing a richer pool of resources and knowledge, they can accelerate the alleviation of external legitimacy pressures, which may amplify the inverted U-shaped relationship between board size and greenwashing [93]; on the other hand, they may enhance oversight by addressing managerial blind spots, thereby reducing greenwashing opportunities and weakening this inverted U-shaped relationship [94]. Therefore, this pattern also falls under type (iii). Since we are unable to predict which mechanism will dominate, we propose a non-directional hypothesis:

H3. Functional background diversity moderates the inverted U-shaped relationship.

#### 3.2.3 Nationality diversity.

Foreign directors also help mitigate two elements of the fraud triangle: opportunity and pressure. Regarding opportunity, as outsiders to the firm, foreign directors maintain a higher degree of independence from senior management [95]. They can transcend the cultural influences prevalent in China—such as guanxi (personal connections), renqing (favor), and mianzi (face)—and maintain a more objective stance [96]. This enables them to oversee management more impartially and strengthens the board’s ability to prevent controlling shareholders or executives from exploiting governance loopholes [97]. Their reputational capital enhances board accountability, and their professional expertise supports more rational judgment, thereby improving detection capabilities and further reinforcing oversight effectiveness [98]. As a result, the likelihood of greenwashing is directly reduced.

In terms of pressure, foreign directors bring diverse perspectives and professional experience [99]. They introduce ethical standards and advanced governance practices from mature systems into Chinese firms, thereby reinforcing corporate governance structures [100] and encouraging broader consideration of social and environmental issues in board decision-making [101]. This diversity facilitates more comprehensive stakeholder evaluation and fosters more balanced decision-making processes [85]. Empirical evidence confirms that greater representation of foreign directors enhances ESG or CSR performance [102,103], effectively easing legitimacy pressures.

Therefore, foreign directors may exert two simultaneous effects. On the one hand, by providing professional expertise, external resources, and extensive social networks, foreign directors may help firms better engage with external stakeholders and more effectively alleviate legitimacy pressures [104,105]. This effect may accelerate the reduction of greenwashing motivation associated with increasing board size, thereby steepening the inverted U-shaped relationship between board size and greenwashing. On the other hand, due to their relatively independent status and stronger willingness to exercise oversight, foreign directors may significantly enhance the board’s monitoring function and reduce managerial opportunities for greenwashing [106]. This may slow the weakening of oversight caused by an expanding board, thus flattening the inverted U-shaped relationship. Therefore, this mechanism also falls under type (iii) among the four moderation mechanism combinations. Given the current uncertainty regarding which of the two effects is more dominant, we propose the following non-directional hypothesis:

H4: Nationality diversity moderates the inverted U-shaped relationship.

#### 3.2.4 Age diversity.

Age diversity mitigates the opportunity component of the fraud triangle but increases pressure. Concerning opportunity, age-homogeneous boards facilitate cognitive convergence as directors with shared historical experiences develop similar perspectives, fostering groupthink [107] and creating opportunities for unchallenged, questionable ESG claims. Conversely, age-diverse boards enhance critical thinking and monitoring effectiveness [108], reducing management’s opportunities for greenwashing practices.

However, the intergenerational conflict resulting from this diversity may lead to disagreements and undermine team cohesion [109]. Empirical studies by Hafsi and Turgut (2013) [110] and Wu et al. (2024) [68] document that such decreased cohesion can diminish ESG performance, consequently increasing external legitimacy pressure on the organization.

Therefore, age diversity may have two distinct effects. On the one hand, age diversity can enhance board oversight by avoiding groupthink, potentially mitigating the oversight weaknesses associated with larger boards and thus reducing opportunities for ESG greenwashing [111]. On the other hand, differences in values and decision-making styles across various age groups may lead to communication and coordination challenges, potentially limiting the board’s ability to swiftly and effectively respond to stakeholder demands, thereby slowing the alleviation of legitimacy pressures [112]. Consequently, this combination aligns with type (iv) among the four moderation mechanism combinations. These two effects collectively flatten the inverted U-shaped relationship between board size and greenwashing behavior. Therefore, we propose the following hypothesis:

H5. Age diversity moderates the inverted U-shaped, making it flatter.

## 4 Research design

### 4.1 Selection of sample

This study investigates A-share listed companies from 2009 to 2023, using two distinct ESG metrics. First, the Bloomberg ESG disclosure score (*ESG*_*dis*)_ was applied to assess the extent of ESG disclosure by firms [113]. This indicator quantifies the amount of disclosed ESG-related content, both positive and negative, on a scale from 0 to 100, but does not directly reflect actual ESG performance [39]. Second, the Huazheng ESG score was utilized to evaluate genuine ESG performance (*ESG*_*per*_), focusing specifically on the implementation and effectiveness of corporate ESG initiatives within China’s unique institutional and market environment [114,115]. The Huazheng score is particularly advantageous for the Chinese context due to its comprehensive coverage, timely updates, and detailed ESG indicators [116].

As Huazheng ESG data became available in 2009 and Bloomberg has not yet released 2024 ESG disclosure scores for most listed companies, the study period spans December 31, 2009, to December 31, 2023. Firm-level data were sourced from the China Stock Market and Accounting Research database. Financial firms, observations with missing key variables, “ST” or “*ST” designated companies, and companies listed for under one year were excluded [117]. All continuous variables were winsorized at the 1% level to limit the impact of outliers. The final dataset includes 13,037 observations across 1,453 listed companies.

### 4.2 Variables measurements

ESG greenwashing (*GW*), our dependent variable, quantifies the divergence between a firm’s ESG disclosures and actual ESG performance [24]. Higher *GW* values indicate greater potential greenwashing activity, reflecting either inflated ESG disclosures relative to performance or diminished performance relative to disclosures [36]. Conversely, *GW* reduction occurs through either moderated disclosure claims or enhanced substantive performance [118]. This conceptualization suggests two fundamental pathways for mitigating greenwashing: implementing supervisory mechanisms to control exaggerated ESG disclosures [10] and establishing responsibility-fulfillment mechanisms to enhance genuine ESG performance in response to legitimacy demands [3]. For measurement consistency, both ESGdis and ESGper were standardized using their respective means ($\overset{―}{{ESG}_{dis}}$ and $\overset{―}{{ESG}_{per}}$) and standard deviations ($\sigma_{dis}$ and $\sigma_{per}$), with the Equation (1) as follows:


$$\;{GW}_{i,t}=\frac{{ESG}_{dis\;i,t}-\overset{―}{{ESG}_{dis}}}{\sigma_{dis}}-\frac{{ESG}_{per\;i,t}-\overset{―}{{ESG}_{per}}}{\sigma_{per}}$$ (1)

Board size (*Bsize*) constitutes our independent variable, measured as the total number of directors (executive, non-executive, and independent) reported in annual corporate disclosures [53,94]. Four dimensions of diversity serve as moderating variables in our analysis: gender, functional background, nationality, and age [96,110,119,120]. These dimensions are examined for their moderating effects on the board size-greenwashing relationship. Table A1 in Appendix A in S1 File presents variable nomenclature, symbols, and measurement specifications.

This study employed several control variables. Corporate governance controls included board meeting frequency (*Meeting*), which strengthens oversight [121], increases transparency, and reduces agency costs [29,122]; CEO duality (*Dual*), where the CEO also chairs the board, potentially weakening checks and balances [123]; and management shareholding ratio (*Mshare*), with higher ratios reducing agency costs, promoting long-term orientation, and diminishing opportunistic behavior [120]. Financial indicators comprised return on assets (*ROA*), as more profitable firms typically demonstrate superior ESG performance and less motivation for greenwashing [9,124], and cash flow ratio (*Cashflow*), since greater financial resources often correlate with better CSR practices [125]. Firm-level characteristics included firm age (*FirmAge*), with established firms typically exhibiting superior ESG disclosure and performance compared to younger counterparts [126], and State-Owned Enterprise status (*SOE*), as private firms face fewer environmental regulations and thus demonstrate greater propensity for greenwashing [24].

### 4.3 Empirical model

In this study, we examined the board size-greenwashing relationship using ordinary least squares regression with a two-way fixed-effects model (year and firm) to analyze our panel data [8]. This approach mitigated potential estimation bias from omitted variables, unobservable firm-specific characteristics, and temporal trends, strengthening causal inferences. We implemented firm-clustered standard errors to account for potential correlations in firm-level error terms, enhancing the robustness of our findings [127]. Our hypotheses were tested using the following model:


$${GW}_{i,t}=\alpha_{\text{0}}+\alpha_{\text{1}}{{Bsize}_{i,t}+\alpha_{\text{2}}{Bsize}_{i,t}^{\text{2}}+\alpha_{\text{3}}Controls}_{i,t}+\varepsilon_{i,t}$$ (2)

where *i* and *t* represent firm and year, respectively; *Bsize* is the explanatory variable, *Bsize²* is its quadratic form; *GW* is the dependent variable; *Controls* are the control variables; and *ε* is the random disturbance term. Equation (2) is used to test the inverted U-shaped relationship between board size and greenwashing (H1).

Building on Equation (2), we introduced moderating variables and their interaction terms, and constructed Equation (3) as follows:


$${GW}_{i,t}=\beta_{\text{0}}+\beta_{\text{1}}{{Bsize}_{i,t}+\beta_{\text{2}}{Bsize}_{i,t}^{\text{2}}+\beta_{\text{3}}{Diversity}_{i,t}+\beta_{\text{4}}{Diversity}_{i,t}\times {Bsize}_{i,t}+{\beta_{\text{5}}{Diversity}_{i,t}\times {Bsize}_{i,t}^{\text{2}}+\beta }_{\text{6}}Controls}_{i,t}+\varepsilon_{i,t}$$ (3)

Where *Diversity* represents four moderating variables: *Female*, *Funback*, *Nationality* and *Age*. These variables are used to examine the moderating effects of different board diversity dimensions on the relationship between board size and greenwashing, corresponding to the tests of H2 through H5.

## 5 Empirical analysis

### 5.1 Descriptive statistics and bivariate analysis

Table 1 presents descriptive statistics for all variables. *GW* exhibited a mean of −0.327, with a standard deviation (SD) of 1.214. Values ranged from −2.862 to 2.723, with a median of −0.348. These metrics indicate moderate greenwashing practices among Chinese enterprises, with substantial variation across firms, consistent with previous findings [120]. *Bsize* ranged from 4 to 18 directors, with a mean of 9.017 (SD = 1.871), reflecting moderate variation in governance structures across sample firms. Table A2 in S1 File displays correlation coefficients and variance inflation factors (VIF) for all variables. VIF values ranged from 1.032 to 1.571, substantially below the conventional threshold of 10 [128], indicating that multicollinearity does not significantly affect our analysis.

**Table 1:** Descriptive statistics.

Symbol	Observations	Mean	SD	Min.	Median	Max
GW	13037	−0.327	1.214	−2.862	−0.348	2.723
Bsize	13037	9.017	1.871	4.000	9.000	18.000
Female	13037	0.135	0.124	0.000	0.111	0.556
Funback	13037	1.541	0.426	0.530	1.554	2.477
Nationality	13037	0.344	0.266	0.000	0.286	1.000
Age	13037	0.142	0.046	0.052	0.137	0.282
Meeting	13037	10.308	4.723	1.000	9.000	58.000
Dual	13037	0.210	0.407	0.000	0.000	1.000
Mshare	13037	6.303	13.591	0.000	0.033	61.512
FirmAge	13037	2.906	0.352	1.792	2.944	3.526
SOE	13037	0.526	0.499	0.000	1.000	1.000
ROA	13037	0.046	0.058	−0.169	0.040	0.220
Cashflow	13037	0.062	0.069	−0.131	0.058	0.264

### 5.2 Regression results

We first conducted a Hausman test, with p-values equal to 0, demonstrating that the fixed-effects model is more appropriate than the random-effects model [129]. Subsequently, we tested the nonlinear relationship between board size and greenwashing, with the results reported in Column 1 of Table 2. The results show that the coefficient of *Bsize* is significantly positive, while the coefficient of *Bsize*^*2*^ is significantly negative, indicating a clear inverted U-shaped relationship between *Bsize* and *GW*, thus supporting H1. The estimated turning point of board size is 11.2 directors, lying within the observed range (4–18), suggesting a meaningful nonlinear relationship. At the 25th percentile of board size (8 directors), adding one director is associated with a 0.36-point increase in greenwashing, whereas at the 75th percentile (9 directors), the same increase leads to a 0.25-point decrease.

**Table 2:** Main regression results.

	1	2	3
Symbol	Baseline	GMM	PSM
Bsize	0.125**	0.498**	0.121**
	(2.57)	(2.31)	(2.43)
Bsize2	−0.006**	−0.021**	−0.005**
	(−2.36)	(−2.27)	(−2.24)
Meeting	0.008***	0.014***	0.008***
	(2.94)	(2.78)	(2.88)
Dual	0.042	3.496*	0.037
	(1.11)	(1.85)	(0.97)
Mshare	0.002	0.004	0.001
	(0.64)	(0.38)	(0.25)
FirmAge	−0.097	0.201	−0.174
	(−0.53)	(1.41)	(−0.92)
SOE	−0.057	0.813**	−0.053
	(−0.78)	(2.22)	(−0.68)
ROA	−0.345	−4.463***	−0.276
	(−1.34)	(−4.07)	(−1.01)
Cashflow	0.659***	6.491***	0.589***
	(4.28)	(3.54)	(3.50)
L.GW		0.499***	
		(16.23)	
Constant	−0.802	−5.411***	−0.494
	(−1.37)	(−3.11)	(−0.81)
Year	Yes	Yes	Yes
Firm	Yes	Yes	Yes
AR (1) p-value		0.000	
AR (2) p-value		0.760	
Hansen test p-value		0.181	
Observations	12,999	11,039	11,134
R-squared	0.653		0.653

Robust t-statistics in parentheses.

*** p < 0.01, ** p < 0.05, * p < 0.1.

Next, we employed two methods to address endogeneity issues. First, we employ the two-step system generalized method of moments (GMM), incorporating lagged values of the dependent variable (*L.GW*) into the regression equation to capture potential dynamic effects, and using appropriate instrumental variables (typically lagged values of independent or control variables) to effectively address reverse causality and potential endogeneity issues [130]. The results in Column 2 of Table 2 confirm the robustness of our GMM model, with a significant AR (1) p-value and non-significant AR (2) and Hansen test p-values. Importantly, the GMM regression results align with the baseline regression results. Second, to address the endogeneity due to sample selection bias, we employed propensity score matching (PSM). Drawing on Shen et al. (2025) [131], we classify firms as treated if their board sizes are at or above the median. For each treated observation, we select its five nearest neighbors from the pool of untreated firms, using all control variables as matching covariates. As a result, the treatment and control groups are comparable across all observable characteristics, except for board size. The matching procedure yields 7,223 treated and 3,969 control observations. The maximum standardized bias among the covariates is 1.5%, well below the recommended 5% threshold [132], indicating satisfactory balance. Table A3 in S1 File reports the balance statistics, confirming that PSM effectively mitigates pre-matching group differences. Table A4 in S1 File presents the average treatment effect on the treated (ATT). Before matching, the difference in greenwashing between the two groups is −0.066; after matching, it reverses to 0.114 and becomes statistically significant (t-value > 2.56). Re-estimating the baseline regression on the matched sample (Table 2, column 3) produces results consistent with the full-sample analysis, reinforcing the conclusion that board size and greenwashing exhibit an inverted U-shaped relationship.

### 5.3 Robustness test

This study employed multiple robustness tests. First, we addressed the possibility of pseudo inverted U-shape relationships using the U test [133]. As shown in Panel A of Table 3, the extreme point (11.2) falls within the observed board size range (4, 18) and is statistically significant (p = 0.0255), confirming the inverted U-shaped relationship.

**Table 3:** Robustness test results.

Panel A: U-test regression result				
U-test		Lower bound		Upper bound
Interval		4		18
Slope		0.0806		−0.0756
t-value		1.95		
P > t		0.0255		
Panel B: Other Robustness Checks				
	1	2	3	4
Symbol	Cubic Regression	GW2	GW3	Extended Controls
Bsize	0.167	0.144**	0.036**	0.114**
	(0.93)	(2.32)	(2.19)	(2.36)
Bsize2	−0.010	−0.006**	−0.002**	−0.005**
	(−0.57)	(−2.07)	(−2.28)	(−2.15)
Bsize3	0.000			
	(0.26)			
Lev				0.432***
				(3.94)
GDP				−0.141
				(−1.49)
Constant	−0.929	−1.146	−0.136	0.635
	(−1.17)	(−1.48)	(−0.66)	(0.56)
Control	Yes	Yes	Yes	Yes
Year	Yes	Yes	Yes	Yes
Firm	Yes	Yes	Yes	Yes
Observations	12,999	12,904	12,999	12,986
R-squared	0.653	0.415	0.394	0.654

Robust t-statistics in parentheses.

*** p < 0.01, ** p < 0.05, * p < 0.1.

Second, we tested for S-shaped relationships by adding a cubic term to our regression [76]. As shown in Panel B, Column 1 of Table 3, the cubic term (*Bsize*^*3*^) is not significant, excluding S-shaped relationships and further supporting the inverted U-shape finding.

Third, we employ two alternative measures of greenwashing. First, to capture more precisely the extent of greenwashing relative to an industry benchmark, we depart from the baseline approach of full-sample standardization. Instead, we normalize ESG disclosure and ESG performance within each industry–year cell and construct an industry-adjusted measure [134], *GW2*. Second, following Chen et al. (2025) [135], we assess firms’ greenwashing behavior by comparing the intensity of ESG rhetoric in the ESG/CSR/ sustainability reports with their actual performance. To measure ESG rhetoric, drawing on Huang et al. (2025) [136], we compile a list of ESG-related keywords (see the supplementary materials in S2 File) and calculate their frequency in the text. We then define a dummy variable *Oral*, which equals 1 if this frequency is above the contemporaneous industry median and 0 otherwise. To capture actual performance, we define another dummy variable *Actual*, which equals 1 if the firm receives an environmental penalty in that year and 0 otherwise. We then construct the alternative greenwashing indicator *GW3*, which equals 1 when *Oral* = 1 and *Actual* = 1, and 0 otherwise. The results reported in Columns 2 and 3 of Panel B in Table 3 are consistent with the baseline regression findings.

Fourth, we added provincial pross regional product (*GDP*) to control for regional economic development disparities [39] and leverage (*Lev*) to account for its potential impact on unethical behavior [9]. These additional controls were introduced at this stage to maintain baseline model parsimony. As shown in Panel B, Column 4, results remain consistent.

Finally, we conducted additional checks by altering standard error clustering to address potential cross-cluster correlations [8] and by replacing firm fixed effects with alternative specifications (industry or regional). These results, consistent with baseline findings, are available in Table A5 in S1 File.

### 5.4 Moderating effects

**Fig 3:**
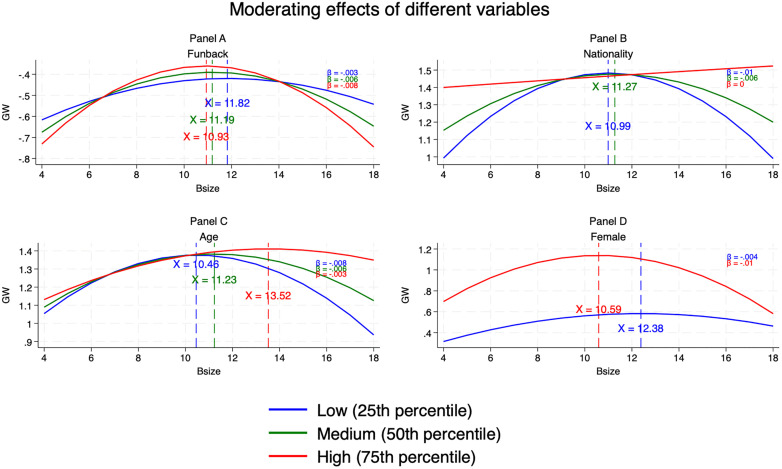
Moderating effects of board diversity dimensions: function, nationality, age, and gender. Note: In Panel D, “Low” indicates Female < 2, and “High” indicates Female ≥ 2.

Table 4 presents the regression estimates for the moderating effects of board diversity on the inverted-U relationship between board size and greenwashing. For gender diversity (Column 1), the interaction term *Bsize×Female*^*2*^ is negative but insignificant, offering no empirical support for H2. For functional background diversity (Column 2), the coefficient on *Bsize×Funback*^*2*^ is significant (β= −0.008, p < 0.10), supporting H3. Its negative sign indicates that the curve becomes steeper, suggesting that the resource provision function outweighs the monitoring function. This dominant effect manifests as a more effective alleviation of legitimacy pressure, ultimately reducing the firm’s motivation for greenwashing [3]. Panel A of Fig 3 plots the predicted curves at the 25th, 50th, and 75th percentiles of functional background diversity, showing that higher functional diversity steepens the inverted-U, reduces the quadratic coefficient, and shifts the turning point leftward.

**Table 4:** Moderating effect regression results.

	1	2	3	4
Symbol	Model1	Model2	Model3	Model4
Bsize	0.044	−0.119	0.273***	0.293**
	(0.68)	(−0.78)	(3.72)	(2.54)
Bsize2	−0.002	0.006	−0.013***	−0.016***
	(−0.59)	(0.83)	(−3.51)	(−2.77)
Female	−3.416**			
	(−1.96)			
Bsize×Female	0.622*			
	(1.73)			
Bsize×Female2	−0.030			
	(−1.61)			
Funback		−0.695		
		(−1.53)		
Bsize×Funback		0.156*		
		(1.73)		
Bsize×Funback2		−0.008*		
		(−1.72)		
Nationality			2.447***	
			(2.96)	
Bsize×Nationality			−0.473***	
			(−2.85)	
Bsize×Nationality2			0.022***	
			(2.70)	
Age				4.918
				(1.25)
Bsize×Age				−1.219
				(−1.56)
Bsize×Age2				0.072*
				(1.91)
Constant	−0.307	0.241	−1.576**	−1.541*
	(−0.49)	(0.26)	(−2.43)	(−1.88)
Control	Yes	Yes	Yes	Yes
Year	Yes	Yes	Yes	Yes
Firm	Yes	Yes	Yes	Yes
Observations	12,999	12,999	12,999	12,999
R-squared	0.654	0.653	0.653	0.653

Robust t-statistics in parentheses.

*** p < 0.01, ** p < 0.05, * p < 0.1.

For nationality diversity (Column 3), the interaction *Bsize×Nationality*^*2*^ is highly significant (β = 0.022, p < 0.01), supporting H4. Its positive sign indicates that the curve becomes flatter, suggesting that supervision—rather than resource provision—is the dominant function for foreign directors, making the reduction of greenwashing opportunities their primary path to curbing greenwashing behavior. Panel B shows that higher nationality diversity flattens the curve until it approaches linearity at the 75th percentile, with the turning point shifting rightward and eventually disappearing.

For age diversity (Column 4), the coefficient on *Bsize×Age*^*2*^ is positive and significant (β = 0.072, p < 0.10), supporting H5. Panel C shows that greater age heterogeneity flattens the inverted-U, enlarges the quadratic term, and shifts the turning point rightward.

Collectively, these findings indicate that demographic and cognitive forms of diversity alter both the magnitude and the location of the non-linear association between board size and greenwashing, albeit in divergent directions depending on the specific diversity dimension.

### 5.5 Test of “critical mass”

Our initial tests found no evidence that gender diversity—measured as the proportion of female directors—moderates the inverted-U relationship between board size and greenwashing. A possible explanation is that proportional measures do not capture the “critical mass” needed for minority directors to exert meaningful influence [137]. Extant research suggests that boards require at least three female directors before observable strategic effects emerge, such as enhanced carbon performance [138] or improved ESG performance [86]. To incorporate this threshold logic, we re-specified gender diversity using three dummy variables [139,140]: *Female_one*, *Female_two*, and *Female_three*, equal to 1 when a board has at least 1, 2, or 3 female directors, respectively. Each dummy was interacted with both the linear and quadratic terms of board size, yielding six interaction terms *Bsize×Female_one*, *Bsize×Female_two*, *Bsize×Female_three* and *Bsize×Female_one*^*2*^, *Bsiz×Female_two*^*2*^, *Bsize×Female_three*^*2*^. Separate regressions were then estimated, and the results appear in Table 5. Columns 2 and 3 show that the coefficients on *Bsize×Female_two*^*2*^ and *Bsize×Female_three*^*2*^ are significantly negative, indicating that boards with at least two female directors exceed the critical-mass threshold and meaningfully moderate the inverted-U. Panel D of Fig 3 illustrates this effect: the quadratic term becomes more negative (from –0.004 to –0.01), the curve steepens, and the turning point shifts leftward (from 10.59 to 12.38). These findings provide partial support for H2. Similarly, as with functional background diversity, the negative coefficient indicates that the resource provision function of female directors outweighs their monitoring function and thus becomes the dominant role. As board size increases, this manifests in a more rapid alleviation of legitimacy pressure.

**Table 5:** Moderating effect of female critical mass.

	(1)	(2)	(3)
Symbol	Model1	Model2	Model3
Bsize	0.047	0.094*	0.111**
	(0.61)	(1.79)	(2.22)
Bsize2	−0.002	−0.004	−0.005**
	(−0.49)	(−1.48)	(−2.07)
Female_one	−0.692*		
	(−1.67)		
Bsize×Female_one	0.120		
	(1.48)		
Bsize×Female_one2	−0.005		
	(−1.42)		
Female_two		−0.600	
		(−1.43)	
Bsize×Female_two		0.121	
		(1.51)	
Bsiz×Female_two2		−0.006*	
		(−1.70)	
Female_three			−1.342*
			(−1.96)
Bsize×Female_three			0.243*
			(1.85)
Bsize×Female_three2			−0.010*
			(−1.71)
Constant	−0.340	−0.635	−0.731
	(−0.52)	(−1.07)	(−1.25)
Control	Yes	Yes	Yes
Year	Yes	Yes	Yes
Firm	Yes	Yes	Yes
Observations	12,999	12,999	12,999
R-squared	0.654	0.653	0.653

Robust t-statistics in parentheses.

*** p < 0.01, ** p < 0.05, * p < 0.1.

## 6 Discussion and conclusion

### 6.1 Research conclusions

This study examined why board size is neither an unqualified problem nor a universal remedy for greenwashing. Integrating FTT with agency and resource dependence perspectives, we theorized and confirmed an inverted-U relationship: greenwashing is lowest when boards are very small or very large and peaks at moderate sizes. Small boards tighten monitoring and restrict opportunities for symbolic disclosure, whereas large boards provide sufficient legitimacy-building resources to ease external pressure to greenwash. At intermediate sizes, opportunity and pressure intersect, generating the highest propensity for symbolic ESG communication. This non-linear pattern refines dominant linear accounts in governance research and shows how FTT can help explain non-linear effects in sustainability outcomes. We further investigated how board diversity conditions this relationship by examining how different diversity dimensions shift the balance between opportunity and pressure.

For gender diversity, we find no significant moderating effect when it is measured as a continuous proportion. Once a critical mass is considered, however, the presence of at least two female directors clearly steepens the inverted-U. This suggests that a relatively low threshold is sufficient for women to exert visible influence in Chinese listed firms and that the often-cited Western benchmark of three female directors may not be universal [141]. Beyond this threshold, our evidence indicates that female directors primarily operate through a resource channel. Because greenwashing is fundamentally a strategy of legitimacy management rather than a purely agency-driven problem [3], external legitimacy resources—such as stakeholder networks, symbolic capital, and credible signals of social commitment [142]—are especially effective in addressing the institutional pressures that give rise to greenwashing. This mechanism is particularly salient in China, where legitimacy pressures from social media and international investors have intensified [51,143], rendering such resources more consequential than marginal improvements in monitoring alone [144].

Functional background diversity produces a similar steepening effect. Heterogeneous functional expertise equips boards with problem-solving capabilities and ESG-relevant knowledge that can improve substantive ESG performance and thereby accelerate the decline in legitimacy pressure as boards grow. In our setting, this performance-enhancing role appears to dominate the supervisory benefits of cognitive diversity, which operate more indirectly by preventing poor decisions ex post. This aligns with the view that domain-specific knowledge and implementation capacity can reduce reliance on symbolic disclosure more quickly than incremental monitoring improvements [90].

By contrast, nationality diversity tends to flatten the inverted-U. Foreign directors’ independence from local relationship networks and guanxi-based ties enhances supervisory effectiveness and reduces opportunities for greenwashing [145]. Yet their ability to supply legitimacy resources is constrained by institutional distance and limited familiarity with local regulatory frameworks, stakeholder expectations, and culturally embedded ESG priorities [146]. Nationality diversity therefore operates mainly through a supervision channel that constrains opportunistic disclosure, rather than through a strong resource channel that rapidly alleviates pressure.

Age diversity displays a more hybrid pattern. Intergenerational heterogeneity can broaden cognitive perspectives and mitigate groupthink [64], strengthening oversight and reducing opportunities for greenwashing. At the same time, age-related value differences and communication frictions may slow consensus-building on ESG strategies and delay performance improvements [147]. This combination dampens both the erosion of opportunity and the relief of pressure, flattening the curve.

Taken together, our findings show that moderately sized boards are most prone to greenwashing and that different diversity dimensions systematically condition this non-linearity. Gender and functional diversity sharpen the inverted-U by enhancing legitimacy-related resources, while nationality diversity primarily tightens supervision and flattens the curve. Age diversity exhibits a hybrid pattern, simultaneously strengthening oversight and slowing pressure relief. These patterns are especially salient in China’s institutional environment, where ESG disclosure is expanding rapidly under evolving regulation, capital markets remain relatively nascent, and firms face complex legitimacy pressures from different stakeholders. In such a setting, boards must continually rebalance opportunity and pressure in response to shifting expectations, rendering both board size and board composition critical levers in shaping greenwashing behavior.

### 6.2 Theoretical implications

This study advances corporate governance theory through three interconnected contributions. First and most fundamentally, we demonstrate how board structural and compositional characteristics interact in nonlinear, directionally opposite ways to shape greenwashing. By transplanting FTT from its traditional fraud detection context to ESG disclosure, we develop and empirically confirm a novel configurational framework: board size exhibits an inverted-U relationship with greenwashing, but this base nonlinearity is systematically reshaped—steepened or flattened—by theoretically distinct diversity dimensions operating through resource-based versus supervision-based mechanisms. This integrated “nonlinear base + dual-mechanism moderation” insight moves beyond traditional linear agency models and isolated diversity effects, revealing board governance as a complex adaptive system where structural and compositional levers function interdependently rather than additively.

Second, our fine-grained decomposition of four diversity attributes challenges the prevailing “surface-level versus deep-level” binary in governance research. Rather than treating diversity as monolithic, we theorize and demonstrate how each dimension—gender, functional background, nationality and age—recalibrates the opportunity–pressure calculus through distinct supervision and resource channels. This attribute-specific approach explains why diversity effects on corporate misconduct are contingent and context-dependent, providing a mechanism-based explanation for the mixed findings that have plagued prior literature.

Third, our finding that a two-woman threshold constitutes critical mass in Chinese boardrooms challenges the Western “three-director” benchmark established in developed markets, underscoring how institutional context modulates the translation of diversity into governance outcomes. This cross-institutional insight highlights the contextual malleability of governance mechanisms and cautions against universal prescriptions in diversity regulation.

Collectively, these contributions recast board structure and composition as interdependent, nonlinear determinants of ESG greenwashing, offering a contingency-based framework that integrates agency theory, resource dependence, and FTT perspectives.

### 6.3 Managerial implications

The findings of this study offer clear guidance for sustainable governance: by strategically designing corporate board structures, organizations can significantly reduce the risk of greenwashing. We propose two evidence-based governance recommendations for enterprises, emphasizing that optimizing board composition and diversity strategies can not only curb misleading sustainability claims but also strengthen genuine ESG governance.

First, optimize board size strategically. Our results demonstrate an inverted U-shaped relationship between board size and greenwashing propensity, with medium-sized boards (10–13 members) exhibiting the highest risk. Organizations should evaluate whether their current board composition falls within this high-risk range and consider either downsizing to enhance accountability or expanding to strengthen legitimacy resources [59]. Although optimal size may vary across organizational contexts, the fundamental objective remains consistent: ensuring boards possess sufficient capacity to either rigorously scrutinize ESG claims or actively promote authentic sustainability performance [8].

**Fig 4:**
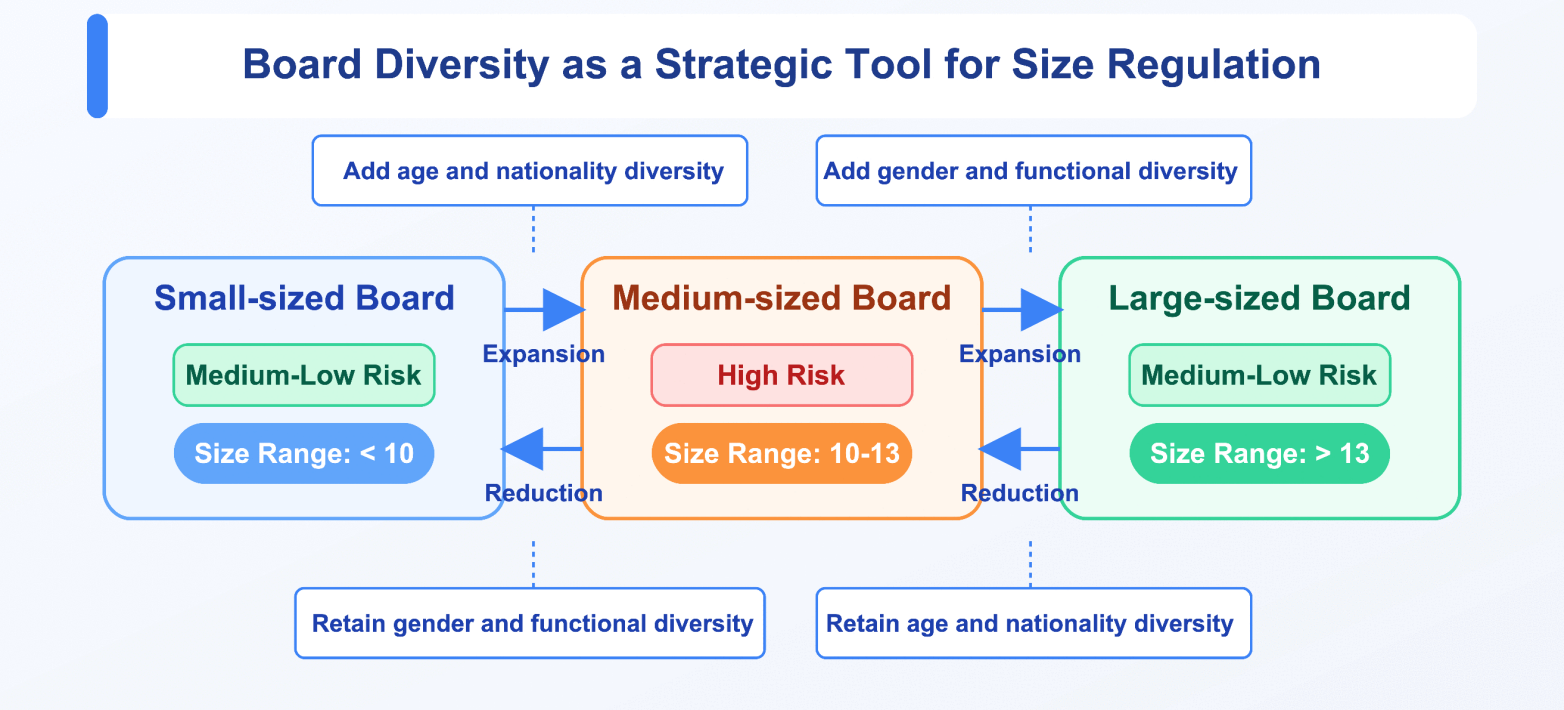
Strategic diversity configurations for managing greenwashing across board sizes.

Second, implement targeted diversity management aligned with board size. As illustrated in Fig 4, when board expansion is necessary, for smaller boards (fewer than 10 members), giving priority to appointing directors with diverse age and nationality backgrounds can effectively delay the rise of greenwashing tendencies. For larger boards (more than 13 members), priority should be given to increasing the number of female directors (to at least two) and directors with diverse functional backgrounds, in order to accelerate the suppression of greenwashing behavior. Similarly, when downsizing the board, smaller boards should retain female directors (ensuring at least two) and those with diverse functional backgrounds to facilitate a faster decline in greenwashing. In contrast, larger boards should retain directors with age and nationality diversity to slow the resurgence of greenwashing risks. This refined approach to diversity management can optimize the governance effect of board size on greenwashing [62].

In conclusion, firms should avoid falling within this high-risk range when designing board size. If this range is unavoidable, they should establish robust governance balancing mechanisms to mitigate potential risks [132].

### 6.4 Limitations and future research

This study has several limitations that point toward promising avenues for future research. First, it focuses solely on Chinese listed companies, limiting generalizability to other institutional contexts. Future cross-national comparative studies could test whether the inverted-U mechanism identified here operates similarly across different regulatory environments, and whether the relative dominance of resource-based versus supervision-based mechanisms varies with institutional factors such as stakeholder activism and cultural orientations toward corporate social responsibility.

Second, our analysis treats the board as homogeneous and does not differentiate among executive, non-executive, and independent directors. These roles vary in responsibilities and incentives, which may shape their contributions to opportunity reduction and pressure alleviation differently. Independent directors may primarily enhance monitoring capacity, while executive directors may be better positioned to mobilize legitimacy resources. Future work using director-level data could examine how the balance between supervision-based and resource-based mechanisms shifts across director categories at different board sizes.

Third, our measurement of greenwashing has several limitations. Our baseline proxy—defined as the difference between normalized ESG disclosure and ESG performance—may be noisy due to measurement error, heterogeneity in rating methodologies, and unobserved firm- and industry-level characteristics. Although we address this concern through robustness checks (e.g., a text-based “talk–walk” measure, GW3), this approach relies on keyword frequencies and therefore cannot distinguish vague, symbolic language from specific, verifiable claims. Future research could improve measurement precision by using natural language processing to classify sentence-level specificity or by constructing indicators that capture the relative weight of quantitative metrics versus qualitative statements in ESG narratives.

Fourth, despite using fixed effects, GMM, and PSM to address endogeneity, residual concerns remain from unobserved time-varying factors. Future research could employ natural experiments—such as regulatory changes mandating board size or diversity thresholds—to strengthen causal inference and test whether the mechanisms underlying the inverted-U relationship align with our theoretical predictions.

Finally, although this study distinguishes between resource-based and supervision-based mechanisms in the moderating roles of gender, functional background, and nationality diversity, it does not make a similar distinction for age diversity, leaving important scope for future research. Future studies could further examine whether the moderating effect of age diversity on the inverted U-shaped relationship between board size and greenwashing is primarily driven by resource-based mechanisms or by supervision-based mechanisms. In addition, future research could investigate the interaction effects among different diversity dimensions to identify board configurations that are more effective in simultaneously constraining opportunity and alleviating pressure.

## Supporting information

S1 Data Data. (XLSX)

S1 Fig Graphical abstract. (PNG)

S1 File Appendix. (DOCX)

S2 File Supplementary materials. (DOCX)
