# Supplementary figures and images for: Optimizing board structure for ESG integrity: Nonlinear size effects and diversity moderation on greenwashing
Source: PLoS One. 2026 Jan 23;21(1):e0335803. doi: 10.1371/journal.pone.0335803 (PMC12829871; doi:10.1371/journal.pone.0335803)

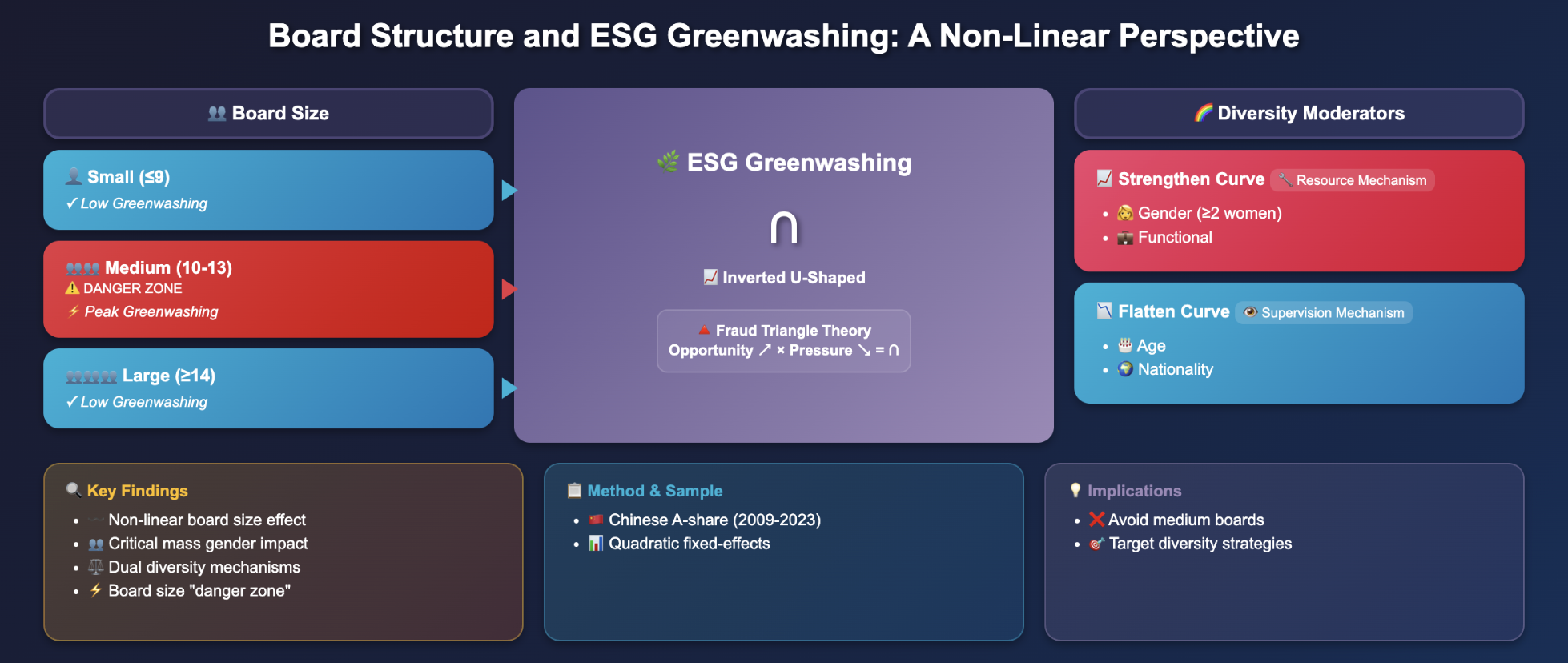

Supplement: S1 Fig — (PNG) [file pone.0335803.s002.png]
