# Supplementary material for: Optimizing board structure for ESG integrity: Nonlinear size effects and diversity moderation on greenwashing
Source: PLoS One. 2026 Jan 23;21(1):e0335803. doi: 10.1371/journal.pone.0335803 (PMC12829871; doi:10.1371/journal.pone.0335803)
Supplement: S1 File — (DOCX) [file pone.0335803.s003.docx]

**Appendix A**

Table A1. Variable descriptions and measurements.

| Variable | Symbol | Measurement |
| --- | --- | --- |
| ESG Greenwashing | GW | The difference between ESG disclosure and ESG performance after normalization |
| ESG Disclosure | ESGdis | Bloomberg’s ESG disclosure score |
| ESG Performance | ESGper | Huazheng ESG score |
| Board Size | Bsize | The number of board members |
| Gender Diversity | Female | The percentage of female board members |
| Functional Background Diversity | Funback | using the Shannon index，$-\sum_{i=1}^{n} p_{i}\times ln\left( p_{i} \right)$; pi represents the proportion of members with the category i functional background in the board; n is the number of types of functional backgrounds in the board. 1 = Production, 2 = R&D, 3 = Design, 4 = Human Resource, 5 = Management, 6 = Marketing, 7 = Finance, 8 = Finance management, 9 = Legal, 99 = Other or unclear occupational areas |
| Nationality Diversity | Nationality | The proportion of foreign directors on the board |
| Age Diversity | Age | using a coefficient of variation $\sigma\div\mu$ |
| Board Meeting | Meeting | The total number of board meetings during the year |
| CEO Duality | Dual | A dummy variable equaling 1 if the CEO is also the chairperson; otherwise, 0 |
| Management Shareholding Ratio | Mshare | The ratio of shares held by senior managers to total shares |
| Return on Assets | ROA | Net profit / total assets ending balance |
| Cash Flow Ratio | Cashflow | Net cash flow from operating activities / total assets |
| Firm Age | FirmAge | The natural logarithm of the current year minus the year of incorporation |
| State-Owned Enterprise | SOE | 1 if state-owned enterprise; otherwise, 0 |

Table A2. Correlation matrix.

|  | VIF | GW | Bsize | Female | Funback | Nationality | Age | Meeting | Dual | Mshare | FirmAge | SOE | ROA | Cashflow |
| --- | --- | --- | --- | --- | --- | --- | --- | --- | --- | --- | --- | --- | --- | --- |
| GW |  | 1 | -0.028*** | 0.064*** | 0.270*** | 0.011 | -0.049*** | 0.075*** | 0.046*** | 0.006 | 0.349*** | -0.063*** | -0.103*** | 0.034*** |
| Bsize | 1.121 | -0.030*** | 1 | -0.126*** | 0.105*** | 0.079*** | 0.000 | -0.024*** | -0.193*** | -0.179*** | 0.001 | 0.256*** | -0.041*** | 0.018** |
| Female | 1.093 | 0.053*** | -0.108*** | 1 | 0.059*** | -0.016* | 0.122*** | 0.019** | 0.114*** | 0.157*** | 0.136*** | -0.216*** | 0.075*** | 0.053*** |
| Funback | 1.061 | 0.267*** | 0.106*** | 0.056*** | 1 | -0.054*** | -0.021** | 0.027*** | 0.015* | 0.023*** | 0.183*** | 0.040*** | -0.016* | 0.040*** |
| Nationality | 1.074 | -0.001 | 0.075*** | -0.012 | -0.066*** | 1 | 0.038*** | 0.032*** | -0.043*** | -0.140*** | 0.144*** | 0.064*** | -0.043*** | -0.006 |
| Age | 1.121 | -0.044*** | -0.013 | 0.122*** | -0.021** | 0.049*** | 1 | 0.006 | 0.105*** | 0.182*** | -0.061*** | -0.294*** | 0.040*** | -0.000 |
| Meeting | 1.030 | 0.051*** | 0.011 | 0.007 | 0.015* | 0.050*** | -0.004 | 1 | 0.026*** | 0.076*** | 0.072*** | -0.039*** | -0.108*** | -0.151*** |
| Dual | 1.141 | 0.042*** | -0.174*** | 0.115*** | 0.017* | -0.037*** | 0.112*** | 0.015* | 1 | 0.274*** | -0.039*** | -0.315*** | 0.095*** | 0.036*** |
| Mshare | 1.414 | -0.028*** | -0.199*** | 0.112*** | 0.014 | -0.207*** | 0.091*** | 0.019** | 0.246*** | 1 | -0.088*** | -0.600*** | 0.223*** | 0.052*** |
| FirmAge | 1.135 | 0.317*** | 0.002 | 0.131*** | 0.170*** | 0.127*** | -0.064*** | 0.068*** | -0.035*** | -0.203*** | 1 | 0.093*** | -0.122*** | -0.017* |
| SOE | 1.559 | -0.061*** | 0.260*** | -0.219*** | 0.042*** | 0.040*** | -0.299*** | -0.025*** | -0.315*** | -0.460*** | 0.093*** | 1 | -0.211*** | -0.087*** |
| ROA | 1.425 | -0.105*** | -0.035*** | 0.065*** | -0.018** | -0.026*** | 0.028*** | -0.093*** | 0.080*** | 0.177*** | -0.108*** | -0.176*** | 1 | 0.500*** |
| Cashflow | 1.388 | 0.037*** | 0.013 | 0.056*** | 0.034*** | -0.004 | 0.007 | -0.134*** | 0.042*** | 0.072*** | -0.012 | -0.098*** | 0.516*** | 1 |

Note: * p< 0.1 ** p<0.05 *** p<0.01; Spearman (upper triangle), Pearson (lower triangle)

Table A3. Balancing test: nearest neighbor matching (1:5) using Bsize as the treatment variable.

| Symbol | Sample | Mean | | Bias(%) | Reduct \|bias\|(%) | t-test | |
| --- | --- | --- | --- | --- | --- | --- | --- |
|  |  | Treated | Control |  |  | t | p>t |
| Meeting | Unmatched | 10.262 | 10.412 | -3.2 | 89.6 | -1.67 | 0.095 |
|  | Matched | 10.263 | 10.278 | -0.3 |  | -0.22 | 0.822 |
| Dual | Unmatched | 0.16298 | 0.3153 | -36.3 | 97.4 | -19.99 | 0.000 |
|  | M Matched | 0.16304 | 0.15903 | 1 |  | 0.73 | 0.464 |
| Mshare | Unmatched | 4.9989 | 9.2553 | -29.5 | 98 | -16.66 | 0.000 |
|  | Matched | 5.0005 | 4.9143 | 0.6 |  | 0.48 | 0.633 |
| FirmAge | Unmatched | 2.9078 | 2.9029 | 1.4 | 91.8 | 0.74 | 0.457 |
|  | Matched | 2.9078 | 2.9073 | 0.1 |  | 0.08 | 0.938 |
| SOE | Unmatched | 0.58337 | 0.39745 | 37.8 | 99.4 | 19.89 | 0.000 |
|  | Matched | 0.58323 | 0.58436 | -0.2 |  | -0.15 | 0.878 |
| ROA | Unmatched | 0.04502 | 0.04881 | -6.4 | 94.9 | -3.44 | 0.001 |
|  | Matched | 0.04503 | 0.04522 | -0.3 |  | -0.23 | 0.820 |
| Cashflow | Unmatched | 0.06137 | 0.06365 | -3.3 | 55.3 | -1.75 | 0.081 |
|  | Matched | 0.06137 | 0.06035 | 1.5 |  | 0.99 | 0.324 |
| Year | Unmatched | 8.7249 | 9.8232 | -27.1 | 95.3 | -14.03 | 0.000 |
|  | Matched | 8.7275 | 8.7796 | -1.3 |  | -0.85 | 0.397 |
| panel_id | Unmatched | 684.33 | 653.29 | 7.7 | 86.7 | 4.04 | 0.000 |
|  | Matched | 684.21 | 688.32 | -1 |  | -0.68 | 0.496 |

Table A4. Average treatment effect on the treated (ATT) from propensity score matching.

| Symbol | Sample | Treated | Controls | Difference | S.E. | T-stat |
| --- | --- | --- | --- | --- | --- | --- |
| GW | Unmatched | -0.346811015 | -0.280898492 | -0.065912523 | 0.023063707 | -2.86 |
|  | ATT | -0.346330198 | -0.460471525 | 0.114141326 | 0.026558803 | 4.30 |
|  | ATU | -0.281231822 | -0.18431927 | 0.096912552 | . |  |
|  | ATE |  |  | 0.108864163 | . |  |

Table A5. Additional robustness test.

|  | Clustered SE | | | Fixed Effects | | |
| --- | --- | --- | --- | --- | --- | --- |
| Symbol | Industry | City | Province | Industry | City | Province |
| Bsize | 0.125*** | 0.125*** | 0.125*** | 0.106** | 0.111** | 0.127** |
|  | (3.26) | (3.04) | (3.08) | (2.28) | (2.22) | (2.50) |
| Bsize2 | -0.006*** | -0.006** | -0.006** | -0.005** | -0.005* | -0.005** |
|  | (-3.09) | (-2.56) | (-2.43) | (-2.03) | (-1.83) | (-2.05) |
| Meeting | 0.008** | 0.008** | 0.008** | 0.014*** | 0.006 | 0.008** |
|  | (2.17) | (2.22) | (2.53) | (4.55) | (1.64) | (2.05) |
| Dual | 0.042 | 0.042 | 0.042 | 0.045 | 0.044 | 0.035 |
|  | (1.22) | (1.32) | (1.32) | (1.26) | (1.12) | (0.89) |
| Mshare | 0.002 | 0.002 | 0.002 | -0.004*** | -0.004*** | -0.004*** |
|  | (0.70) | (0.73) | (0.73) | (-3.27) | (-2.83) | (-3.11) |
| FirmAge | -0.097 | -0.097 | -0.097 | 0.108* | -0.057 | -0.033 |
|  | (-0.51) | (-0.42) | (-0.43) | (1.93) | (-0.80) | (-0.49) |
| SOE | -0.057 | -0.057 | -0.057 | -0.161*** | -0.090* | -0.106** |
|  | (-0.75) | (-0.79) | (-0.81) | (-4.12) | (-1.91) | (-2.42) |
| ROA | -0.345 | -0.345 | -0.345 | -2.026*** | -1.875*** | -2.171*** |
|  | (-1.19) | (-1.33) | (-1.62) | (-7.93) | (-6.62) | (-7.67) |
| Cashflow | 0.659*** | 0.659*** | 0.659*** | 0.593*** | 1.106*** | 1.244*** |
|  | (4.24) | (4.39) | (3.97) | (3.32) | (5.56) | (6.07) |
| Constant | -0.802 | -0.802 | -0.802 | -1.182*** | -0.753** | -0.912*** |
|  | (-1.39) | (-1.12) | (-1.20) | (-4.23) | (-2.41) | (-2.92) |
| Year Fixed Effects | Yes | Yes | Yes | Yes | Yes | Yes |
| Firm Fixed Effects | Yes | Yes | Yes | No | No | No |
| Clustered by Firm | No | No | No | Yes | Yes | Yes |
| Observations | 12,999 | 12,999 | 12,999 | 13,037 | 13,031 | 13,037 |
| R-squared | 0.653 | 0.653 | 0.653 | 0.441 | 0.421 | 0.374 |

Robust t-statistics in parentheses

*** p<0.01, ** p<0.05, * p<0.1
