# Supplementary material for: Optimizing board structure for ESG integrity: Nonlinear size effects and diversity moderation on greenwashing
Source: PLoS One. 2026 Jan 23;21(1):e0335803. doi: 10.1371/journal.pone.0335803 (PMC12829871; doi:10.1371/journal.pone.0335803)
Supplement: S2 File — (DOCX) [file pone.0335803.s004.docx]

**Extended ESG Chinese dictionary**

**E Environment keywords**

Environment, Environmental protection, Environmental protection (short form), Environmental quality, Environmental governance, Environmental management, Green development, Environmental impact, Environmental pollution, Environmental accidents, Environmental incidents, Environmental risk, Environmental emergency response plan, Environmental protection investment, Environmental protection investment (short form), Environmental early warning, Environmental training, Climate change, Climate risk, Climate adaptation, Greenhouse gases, Greenhouse gas emissions, Carbon neutrality, Low-carbon development, Carbon reduction, Carbon abatement, Carbon sink, Carbon credit, Carbon trading, Green finance, Transition plan, Emission reduction, Emission reduction practices, Emission reduction measures, Emission reduction targets, Emission scope, Carbon emissions, Physical risk, Transition risk, Ecosystem, Ecological protection, Biodiversity, Species, Plants, Botany, Soil and water conservation, Ecological restoration, Ecological recovery, Ecological remediation, Ecological governance, Ecological functions, Ecological environment, Ecological civilization, Energy use, Total energy consumption, Total energy consumption, Energy efficiency and resource conservation, Low energy consumption, Energy conservation, Energy conservation targets, Green energy, Clean energy, Energy mix, Energy management, Energy intensity, Energy intensity, Energy efficiency, Energy efficiency, Energy efficiency, Resource use, Resource use, Resource consumption, Resource management, Raw materials, Materials, Packaging materials, Green office, Electricity saving, Resource conservation, Saving resources, Resource saving, Resource recycling, Circular economy, Technological improvement, Renewable resources, Water resources, Water consumption, Water use, Water saving, Recycling, Reuse and recycling, Circular use, Comprehensive utilization, Utilization rate, Positive cycle, Pollutant emissions, Pollution prevention and control, Emissions disclosure, Pollution control facilities, Wastewater treatment, Administrative penalties, Excessive emissions, Emission intensity, Emission concentration, Waste management, Waste recycling, Waste recycling, Wastewater, Waste gas, Waste residue, Solid waste, Solid waste, Solid waste (abbr.), Hazardous waste, Hazardous waste (HW), Harmful waste, Non-hazardous waste, Harmless disposal

⸻

**S Social keywords**

Social responsibility, Rural revitalization, Poverty alleviation, Poverty reduction, Poverty alleviation, Targeted poverty assistance, Support for the poor, Support, Assistance, People’s livelihood and well-being, People’s livelihood, Benefiting the people, Inclusive benefit, Common prosperity, Rural development, Local employment, Local employment, Jobs, Tax payment, Social contribution, Social benefits, Social welfare, Public welfare and charity, Donation, Public utilities, Voluntary activities, Community development, Community relations, Community building, Regional development, Belt and Road Initiative, Belt and Road, Response to national strategies, Technological innovation, Scientific and technological innovation, Commercialization of scientific and technological achievements, R&D innovation, R&D innovation, R&D innovation, R&D investment, R&D investment, R&D investment, R&D personnel, R&D personnel, R&D personnel, Innovation achievements, Utility model patents, Intellectual property, High and new technology, Innovation capability, Innovation management, Digital transformation, Informatization development, Artificial intelligence, Technological cooperation, Technology ethics, Science and technology ethics, Employee rights and interests, Compensation incentives, Employee benefits, Working conditions, Social insurance, Labor disputes, Employment policy, Employment policy, Employee promotion, Human capital development, Human capital development, Employee training, Career development, Talent cultivation, Occupational health, Labor protection, Work safety, Safety management, Employee care, Employee care, Employee satisfaction, Equal opportunity, Diversity, Human rights, People-oriented, Democratic management, Creditors’ interests, Customers, Consumers, Suppliers, Supply chain management, Supply chain risk management, Supply chain security, Supply chain stability, Upstream and downstream, Distributors, Contractors, Agents, Value chain, Industrial chain, Partners, Partnerships, Customer relationships, Small and medium-sized enterprises (SMEs), Product quality, Product recall system, Quality control, After-sales service, Customer complaints, Customer satisfaction, Quality management, Quality certification, Quality testing, Data security, Customer privacy protection, Information security

⸻

**G Governance keywords**

Stakeholders, Stakeholder communication, Information communication, Information transparency, Communication channels, Communication frequency, Reliability of information disclosure, Timeliness of information disclosure, Quality of information disclosure, Sustainability information disclosure, Sustainable development governance, Sustainable development committee, ESG committee, Corporate social responsibility committee, ESG risk management committee, ESG management system, Sustainable development strategy, Sustainable risks and opportunities, Long-term development strategy, Strategic planning, Development prospects, Due diligence, Compliance management, Professional capability, Standardized operation, Performance evaluation, Scope of functions, Organizational structure, Governance structure, Governance mechanism, Ownership structure, Shareholders’ rights, Protection of shareholders’ rights and interests, Shareholder participation, Protection of minority shareholders, Interests of small and medium investors, Investor relations management, Investor protection, Board independence, Board diversity, Board diversity, Management compensation, Compensation system, Compensation framework, Compensation management body, Compensation management system, Incentive and restraint mechanism, Risk management, Risk identification, Risk response, Risk response, Risk control, Risk monitoring, Risk monitoring, Risk management and control, Risk control and management, Internal control, Internal control system, Internal control body, Checks and balances, Internal audit, Audit institution, Audit committee, Audit, Inspection, Verification, Supervision, Board of supervisors, Supervisory body, Supervision system, Accountability mechanism, Accountability system, Reporting, Reporting system, Business ethics, Business ethics, Anti-commercial bribery, Anti-fraud, Anti-fraud, Anti-corruption, Integrity, Anti-money laundering, Professional ethics, Trade secrets, Confidential business information, Whistleblower protection, Integrity, Honesty and trustworthiness, Corporate reputation, Business reputation, Responsible marketing, Tax transparency, Tax transparency, Tax revenue transparency, Fair competition, Fair trading, Fair trade, Insider trading, Related-party transactions, Anti-unfair competition, Anti-monopoly
